# Evaluation of accordance of antibiotics package size with recommended treatment duration of guidelines for sore throat and urinary tract infections

**DOI:** 10.1186/s13756-019-0495-5

**Published:** 2019-02-11

**Authors:** Doris Rusic, Josko Bozic, Josipa Bukic, Ana Seselja Perisin, Dario Leskur, Darko Modun, Sinisa Tomic

**Affiliations:** 1 Department of Pharmacy, University of Split School of Medicine, Soltanska 2, Split, Croatia; 2 Department of Pathophysiology, University of Split School of Medicine, Soltanska 2, Split, Croatia; 3 Agency for Medicinal Products and Medical Devices, Ksaverska cesta 4, Zagreb, Croatia

**Keywords:** Antibiotic resistance, Prescribing guideline, Sore throat, Urinary tract infections

## Abstract

**Background:**

The aim of this study was to investigate whether marketed antibiotics package sizes are in accordance with treatment durations recommended in guidelines for prescribing antibiotics in sore throat and urinary tract infections.

**Methods:**

National drug database was searched with limitation to Antibacterials for systemic use. Formulations which did not have pre-specified dosage unit by the manufacturer were excluded (e.g. powders for oral solutions). The final list contained 94 drugs with 23 different active substances. This list was then cross-referenced with selected antimicrobial prescribing guidelines provided by Intersectoral Society for Antibiotic Resistance Control (ISKRA), National Institute for Health and Care Excellence (NICE) and The Infectious Diseases Society of America (IDSA).

**Results:**

Seven packages matched ISKRA guidelines on sore throat while 16 were mismatched. Considering drug packages under reimbursement, 3 matched ISKRA guidelines and 8 were mismatched. Only 3 packages matched IDSA guidelines for comparable indications, and 18 were mismatched. When considering NICE guidelines there were 5 mismatched and only one package that was in accordance with the guidelines. ISKRA guidelines for urinary tract infections matched 23 packages and mismatched 58 packages. IDSA guidelines for urinary tract infections matched one package and were mismatched in 15 cases.

**Conclusions:**

One of the causes of leftover antibiotics is poor accordance of antibiotic package size with treatment recommendation duration. This should be identified as a potential target for reduction of excess antibiotics in the community. Measures that promote patient adherence to therapy and patient education should be considered essential to manage proper handling of leftover antibiotics.

## Background

Antibiotic resistance is recognized as a global threat to the health care system that may dramatically set back the modern medicine [1]. Various contributors to the emergence of antibiotic resistance have been identified. These include genetic factors intrinsic to bacteria, inappropriate antibiotic prescribing and sales, leftover antibiotics, use of antibiotics outside healthcare sector as well as the high amount of antibiotics released into the wastewater [2, 3]. Furthermore, a positive association has been established between resistance and antibiotic consumption [4].

World Health Organization has released Global Action Plan on Antimicrobial Resistance which includes optimisation of the use of antimicrobial agents [5]. However, global consumption of antibiotics is increasing [6]. From 2000 to 2010 there were estimated 1.4 bilion outpatient antibiotics in the US [7]. Furthermore, estimates are that in 2013 antibiotic usage in China was 162000 tons, 48% of which was human consumption [8].

Antibiotic stewardship programmes have been established as means for antibiotic resistance management and rationalisation of antibiotic consumption. Antibiotic stewardship objectives include, but are not limited to, prescribing empirical therapy according to the guidelines, change to narrow-spectrum antibiotic when culture results are available, presence of a local antibiotic guide and list of restricted antibiotics. An outpatient antimicrobial stewardship intervention, consisting of clinician education may significantly reduce off-guideline antibiotic use [9]. However, previous studies have raised concerns that antibiotics prepacked by the manufacturer may limit prescribers ability to follow antibiotic prescribing guidelines [10, 11]. Improper duration of treatment with antibiotics and leftover excess units of antibiotics may contribute to antibiotic resistance and may increase antibiotic consumption [12]. Sore throat and urinary tract infections are among top 5 leading indications that account for most antibiotic prescribing in primary care [13]. Therefore, the aim of this study was to investigate whether marketed package sizes of antibiotics are in accordance with treatment regimens and durations recommended in national and international guidelines for prescribing antibiotics in sore throat and urinary tract infections.

## Methods

**Fig. 1:**
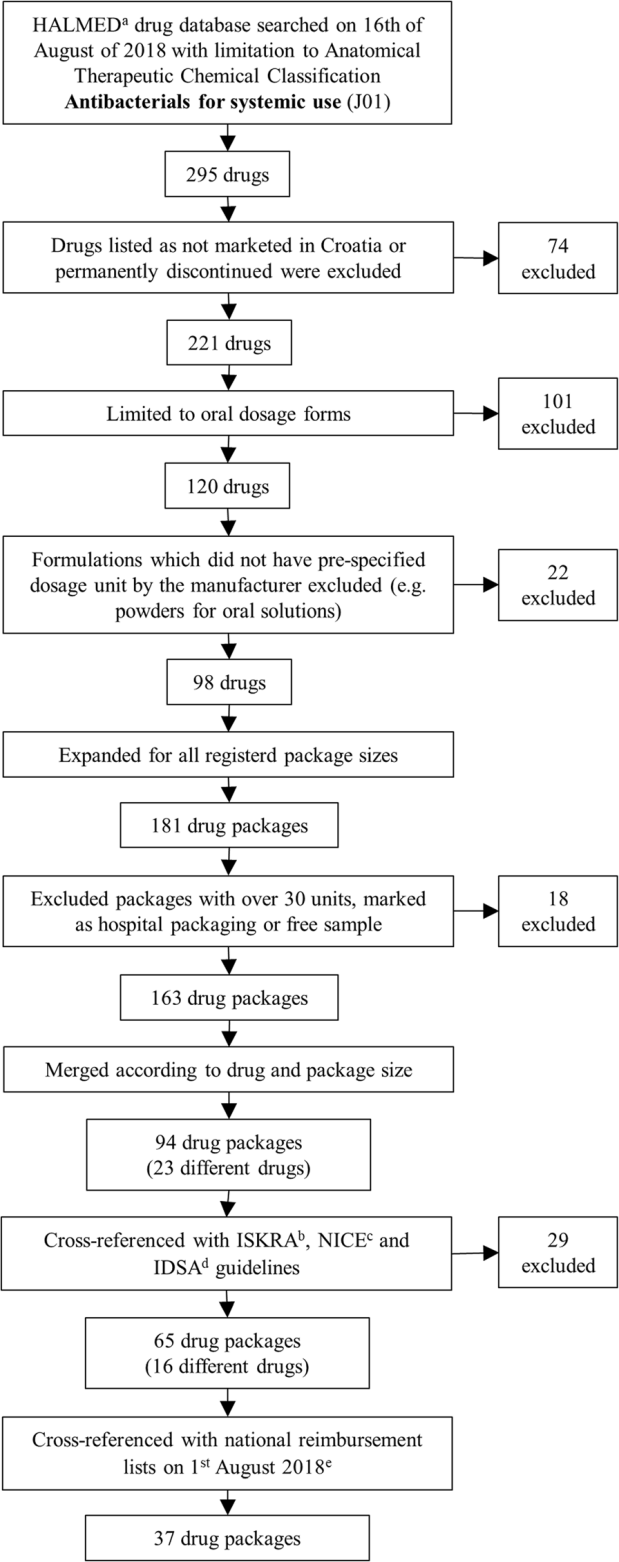
Flowchart of identifying drugs eligible for inclusion in the study. ^a^Croatian Agency for Medicinal Products and Medical Devices. ^b^The Intersectoral Society for Antibiotic Resistance Control. ^c^National Institute for Health and Care Excellence. ^d^Infectious Diseases Society of America. ^e^Available at: http://www.hzzo.hr/zdravstveni-sustav-rh/trazilica-za-lijekove-s-vazecih-lista/, last accessed: 16th August 2018

The Croatian national drug database was searched with limitation to Anatomical Therapeutic Chemical Classification (ATC) Antibacterials for systemic use or J01. This search resulted in 295 drugs (on 16^th^ of August of 2018) exported in a spreadsheet [14]. All drugs listed as not marketed in Croatia or permanently discontinued were excluded from the list. The aim of this study required evaluation of antibiotics regularly prescribed in the primary care and ambulatory setting or antibiotics that are self-administered by the patient. Therefore, the list was further limited to only oral dosage forms. Due to the diversity of dosing (relative to weight of the patient) paediatric formulations were not be considered in this study. All formulations of antibiotics which did not have pre-specified dosage unit by the manufacturer were excluded (e.g. powders for oral solutions). As all registered package sizes of a single trade-name drug were presented as a single result in the exported spreadsheet, the list was further expanded to all possible package sizes and packages with 30 or more units were excluded [14]. Listed drugs were merged based on their active substance, strength and package size, meaning that drugs that had the same active substance in the same strength and with same package size were considered as a single result irrelevant of their trade-name. The final list contained 94 drug packages with 23 drugs. Furthermore, drugs that were included in national reimbursements lists (from 29^th^ of November 2012 to 1^st^ of August 2018), both full and partial, were identified. Detailed workflow chart is presented in Fig. 1.

This list was then cross-referenced with selected antimicrobial prescribing guidelines. The national guidelines are provided by the Intersectoral Society for Antibiotic Resistance Control (ISKRA). Furthermore, other comparable guidelines were consulted. Namely, National Institute for Health and Care Excellence (NICE) antimicrobial prescribing guidelines and The Infectious Diseases Society of America guidelines. Five eligible guidelines were cross-referenced with the final list of drugs:

1. ISKRA guidelines on sore throat: diagnostic and therapeutic approach [15],
2. NICE Sore throat (acute): antimicrobial prescribing [16],
3. Clinical Practice Guideline for the Diagnosis and Management of Group A Streptococcal Pharyngitis: 2012 Update by the Infectious Diseases Society of America [17],
4. ISKRA guidelines on antimicrobial treatment and prophylaxis of urinary tract infections [18],
5. International Clinical Practice Guidelines for the Treatment of Acute Uncomplicated Cystitis and Pyelonephritis in Women: A 2010 Update by the Infectious Diseases Society of America and the European Society for Microbiology and Infectious Diseases [19].

When cross-referencing the list of drugs with the guidelines, comparison was considered only if the guideline specifically indicated the name of the drug, dosage strength, dosing regimen, and duration of treatment. General recommendation for a class of antibiotics was not considered appropriate for this study and was therefore not discussed. Additionally, if a guideline indicated a flexible interval (i.e. 10-14 days) as recommended treatment duration for a specific drug, duration of treatment that would result in least extra units of the drug was considered. It should be noted that some of the guidelines cover various indications and as such suggest different treatment durations all of which were taken into consideration. Recommendations for prophylaxis were not considered. If for a specific drug dosage was provided as mg/kg/dose, maximum dose was considered, and where the dose was provided as a range, dose that would result in least extra units of the drug was considered.

Drug packages with 7 different active ingredients could not be cross referenced with any of the included guidelines. For these drugs accordance was considered with recommendations listed in the Summary of product characteristics (SmPC).

When calculating excess units of antibiotics, 100% adherence to the treatment was assumed. Drugs that would in case of adherence to the regimen proposed by the guidelines result in 0 excess units were considered matched with guidelines. The results are presented as a minimal number of packages needed to complete the course of drug indicated in the guideline and as number of extra units of the drug that are left over after completing this course. Doses of drugs are written in milligrams (mg) or international units (IU) where applicable. Formulations that could be divided in two equal doses allowed analysis when guideline recommended lower dose strength than the formulation. Extra units of such drugs are presented with reference to the original formulation.

## Results

**Fig. 2:**
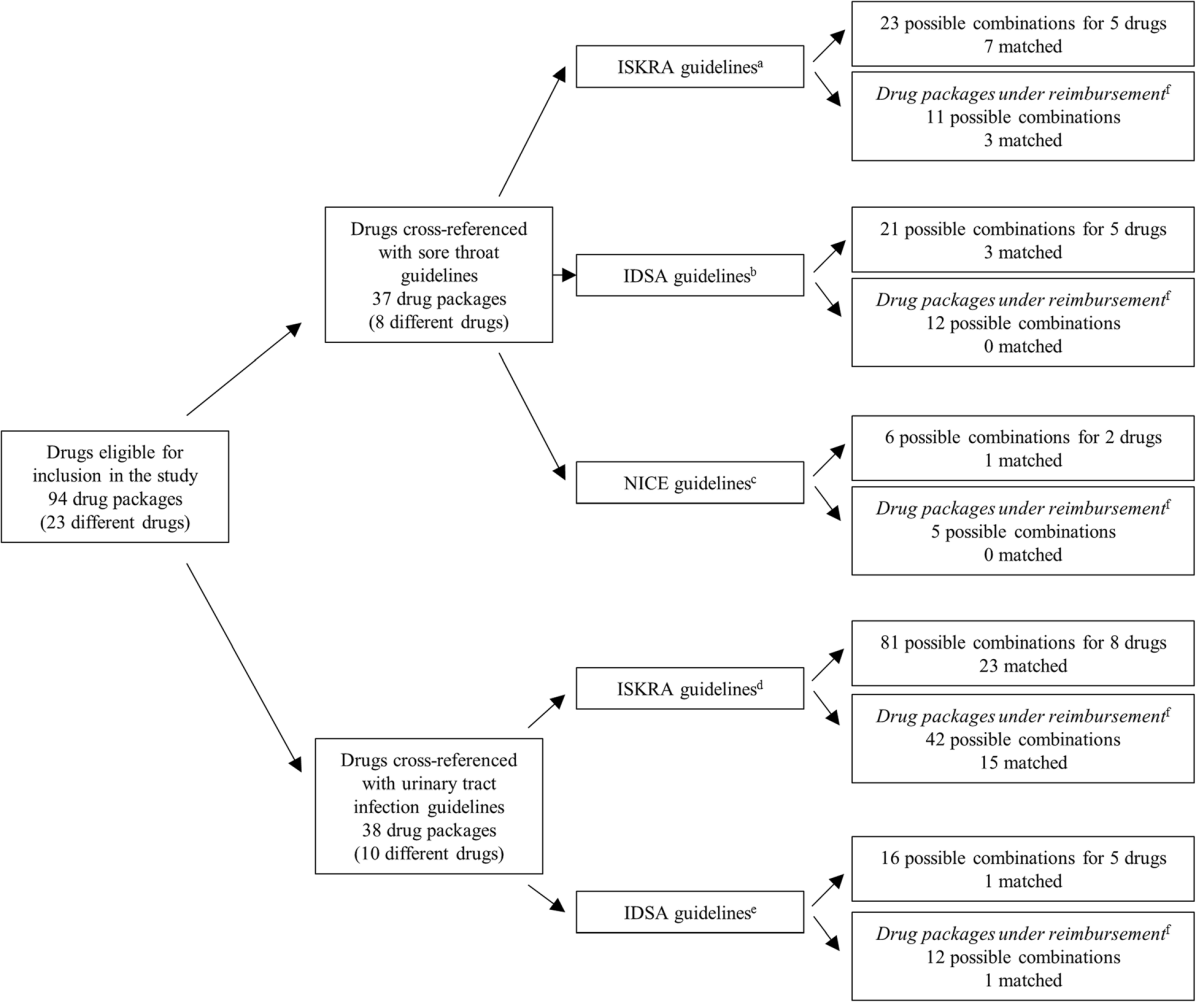
Number of drug packages that would in case of accordance with the regimen proposed by the selected guidelines result in 0 excess units (matched). ^a^The Intersectoral Society for Antibiotic Resistance Control guidelines on sore throat: diagnostic and therapeutic approach and guidelines on antimicrobial treatment and prophylaxis of urinary tract infections. ^b^Clinical Practice Guideline for the Diagnosis and Management of Group A Streptococcal Pharyngitis: 2012 Update by the Infectious Diseases Society of America and International Clinical Practice Guidelines for the Treatment of Acute Uncomplicated Cystitis and Pyelonephritis in Women: A 2010 Update by the Infectious Diseases Society of America and the European Society for Microbiology and Infectious Diseases. ^c^National Institute for Health and Care Excellence Sore throat (acute): antimicrobial prescribing. ^d^The Intersectoral Society for Antibiotic Resistance Control (ISKRA) guidelines on antimicrobial treatment and prophylaxis of urinary tract infections. ^e^International Clinical Practice Guidelines for the Treatment of Acute Uncomplicated Cystitis and Pyelonephritis in Women: A 2010 Update by the Infectious Diseases Society of America and the European Society for Microbiology and Infectious Diseases. ^f^included in Croatian national reimbursement lists on 1^st^ of August 2018

In this study 7 packages matched ISKRA guidelines on sore throat (Fig. 2) while 16 would inevitably result in extra units of antibiotics. Considering drug packages under reimbursement, 3 matched ISKRA guidelines (Fig. 2) and 8 were mismatched. For 5 ISKRA treatment recommendations for 5 different drugs, 3 were matched (2 under reimbursement) (Tables 1 and 2). Only 3 packages matched IDSA guidelines for comparable indications, and 18 were mismatched. None of the matched drug packages were under reimbursement (Fig. 2). From 6 considered IDSA treatment recommendations for 5 different drugs, 3 were matched for 2 drugs as presented in Tables 1 and 2. When considering NICE guidelines there were 5 mismatched and only one package that fully aligned with the guidelines (Fig. 2). Overall, matched drugs were 20x500 mg amoxicillin, 10x1000 mg and 20x1000 mg amoxicillin with clavulanic acid, 6x125 mg, 6x250 mg, 1x500 mg and 3x500 mg azithromycin, 5x500 mg clarithromycin and 30x1500000 IU of penicillin V. There were 5 considered sore throat treatments proposed by ISKRA, 3 of which were matched. Furthermore, 3 out of 6 proposed IDSA treatments and 1 of 3 proposed NICE treatment regimens were matched (Tables 1 and 2).

**Table 1:** Accordance of the package sizes of selected drugs with guidelines for sore throat I

Drug	Package size	ISKRA^a^	Min No. of Packages	Extra units	IDSA^b^	Min No. of Packages	Extra units	NICE^c^
Amoxicillin	16x500 mg^d^	n/a			2x500 mg, 10 days	2	12	n/a
					1x1000 mg, 10 days	2	12	
	20x500 mg	n/a			2x500 mg, 10 days	1	0	
					1x1000 mg, 10 days	1	0	
	16x1000 mg^d^	n/a			2x500 mg, 10 days	1	6	
					1x1000 mg, 10 days	1	6	
	20x1000 mg^e^	n/a			2x500 mg, 10 days	n/a	n/a	
					1x1000 mg, 10 days	1	10	
Co-amoxiclav	10x1000 mg	2x1000 mg, 10 days	2	0	n/a			n/a
	12x1000 mg^d^	2x1000 mg, 10 days	2	4	n/a			
	14x1000 mg^d^	2x1000 mg, 10 days	2	8	n/a			
	16x1000 mg	2x1000 mg, 10 days	2	12	n/a			
	20x1000 mg	2x1000 mg, 10 days	1	0	n/a			
	21x1000 mg	2x1000 mg, 10 days	1	1	n/a			
	24x1000 mg	2x1000 mg, 10 days	1	4	n/a			
	30x1000 mg	2x1000 mg, 10 days	1	10	n/a			
Azithromycin	6x125 mg	1x500 mg, 3 days	2	0	1x500 mg, 5 days	4	4	n/a
	6x250 mg^d^	1x500 mg, 3 days	1	0	1x500 mg, 5 days	2	2	
	1x500 mg	1x500 mg, 3 days	3	0	1x500 mg, 5 days	5	0	
	2x500 mg	1x500 mg, 3 days	2	1	1x500 mg, 5 days	3	1	
	3x500 mg^d^	1x500 mg, 3 days	1	0	1x500 mg, 5 days	2	1	
	6x500 mg	1x500 mg, 3 days	1	3	1x500 mg, 5 days	1	1	
	12x500 mg	1x500 mg, 3 days	1	9	1x500 mg, 5 days	1	7	
	24x500 mg	1x500 mg, 3 days	1	21	1x500 mg, 5 days	1	19	
	1x1000 mg^d^	1x500 mg, 3 days	n/a	n/a	1x500 mg, 5 days	n/a	n/a	
	2x1000 mg	1x500 mg, 3 days	n/a	n/a	1x500 mg, 5 days	n/a	n/a	
	3x1000 mg	1x500 mg, 3 days	n/a	n/a	1x500 mg, 5 days	n/a	n/a	
	6x1000 mg	1x500 mg, 3 days	n/a	n/a	1x500 mg, 5 days	n/a	n/a	
Cephalexin	16x500 mg^d^	n/a			2x500 mg, 10 days	2	12	n/a
	16x1000 mg^d^	n/a			2x500 mg, 10 days	n/a	n/a	
Clindamycin	16x150 mg^d^	3x300 mg, 10 days	4	4	3x300 mg, 10 days	4	4	n/a
	16x300 mg^d^	3x300 mg, 10 days	2	2	3x300 mg, 10 days	2	2	
	16x600 mg^d^	3x300 mg, 10 days	1	1	3x300 mg, 10 days	1	1	
	32x600 mg^d^	3x300 mg, 10 days	1	17	3x300 mg, 10 days	1	17	

Co-amoxiclav – amoxicillin and clavulanic acid; n/a – not applicable; extra units of drugs are presented with reference to the original formulation

^a^The Intersectoral Society for Antibiotic Resistance Control (ISKRA) guidelines on sore throat: diagnostic and therapeutic approach – Croatian national guidelines

^b^Clinical Practice Guideline for the Diagnosis and Management of Group A Streptococcal Pharyngitis: 2012 Update by the Infectious Diseases Society of America

^c^National Institute for Health and Care Excellence (NICE) Sore throat (acute): antimicrobial prescribing

^d^included in Croatian national reimbursement lists on 1^st^ of August 2018

^e^included in Croatian national reimbursement lists prior to 1^st^ of August 2018 (from 29^th^ of November 2012)

**Table 2:** Accordance of the package sizes of selected drugs with guidelines for sore throat II

Drug	Package size	ISKRA^a^	Min No. of Packages	Extra units	IDSA^b^	Min No. of Packages	Extra units	NICE^c^	Min No. of Packages	Extra units
Clarithromycin	14x250 mg^d^	2x250 mg, 10 days	2	8	2x250 mg, 10 days	2	8	2x250-500 mg, 5 days	1	4
	5x500 mg	2x250 mg, 10 days	n/a		2x250 mg, 10 days	n/a		2x250-500 mg, 5 days	2	0
	7x500 mg^d^	2x250 mg, 10 days	n/a		2x250 mg, 10 days	n/a		2x250-500 mg, 5 days	2	4
	14x500 mg^d^	2x250 mg, 10 days	n/a		2x250 mg, 10 days	n/a		2x250-500 mg, 5 days	1	4
Penicillin V	30x1000000 IU^d^	3x1 500 000 IU, 10 days	2	15	4x250 mg, 10 days	n/a		4x500 mg, 5-10 days	n/a	
					2x500 mg, 10 days			2x1000 mg, 5-10 days		
	30x1500000 IU^d^	3x1 500 000 IU, 10 days	1	0	4x250 mg, 10 days	n/a		4x500 mg, 5-10 days	n/a	
					2x500 mg, 10 days			2x1000 mg, 5-10 days		
Erythromycin	16x250 mg^d^	n/a			n/a			4x250-500 mg, 5 days	3	8
								2x500-1000 mg, 5 days	3	8

n/a – not applicable; IU-international unit; extra units of drugs are presented with reference to the original formulation

^a^The Intersectoral Society for Antibiotic Resistance Control (ISKRA) guidelines on sore throat: diagnostic and therapeutic approach – Croatian national guidelines

^b^Clinical Practice Guideline for the Diagnosis and Management of Group A Streptococcal Pharyngitis: 2012 Update by the Infectious Diseases Society of America

^c^National Institute for Health and Care Excellence (NICE) Sore throat (acute): antimicrobial prescribing

^d^included in Croatian national reimbursement lists on 1^st^ of August 2018

ISKRA guidelines for urinary tract infections matched 23 packages and mismatched 58 packages (Fig. 2). From 17 treatments for 8 different drugs recommended by ISKRA, 8 could treatments not be matched with any of the packages (Tables 3 and 4). IDSA guidelines for urinary tract infections matched one package under reimbursement and were mismatched in 15 cases (Fig. 2). From 6 considered treatment regimens in IDSA guidelines, only one could be matched (Table 3). Overall, 5 out of 8 amoxicillin with clavulanic acid packages could be matched, 8 out of 10 cefuroxime, one fosfomycin and both ciprofloxacin and both cefixime packages were matched (Tables 3 and 4).

**Table 3:** Accordance of the package sizes of selected drugs with guidelines for urinary tract infections I

Drug	Package size	ISKRA^a^	Min No. of packages	Extra units	IDSA^b^
Co-amoxiclav	10x1000 mg	2x1000 mg, 7 days	2	6	n/a
		2x1000 mg, 10-14 days	2 (10 days)	0	
		2x1000 mg, 14 days	3	2	
		2x1000 mg, 28 days	6	4	
	12x1000mg^c^	2x1000 mg, 7 days	2	10	
		2x1000 mg, 10-14 days	2 (12 days)	0	
		2x1000 mg, 14 days	3	8	
		2x1000 mg, 28 days	5	4	
	14x1000 mg^c^	2x1000 mg, 7 days	1	0	
		2x1000 mg, 10-14 days	2 (14 days)	0	
		2x1000 mg, 14 days	2	0	
		2x1000 mg, 28 days	4	0	
	16x1000 mg	2x1000 mg, 7 days	1	2	
		2x1000 mg, 10-14 days	2 (14 days)	4	
		2x1000 mg, 14 days	2	4	
		2x1000 mg, 28 days	4	8	
	20x1000 mg	2x1000 mg, 7 days	1	6	
		2x1000 mg, 10-14 days	1 (10 days)	0	
		2x1000 mg, 14 days	2	12	
		2x1000 mg, 28 days	3	4	
	21x1000 mg	2x1000 mg, 7 days	1	7	
		2x1000 mg, 10-14 days	1 (10 days)	1	
		2x1000 mg, 14 days	2	14	
		2x1000 mg, 28 days	3	7	
	24x1000 mg	2x1000 mg, 7 days	1	10	
		2x1000 mg, 10-14 days	1 (12 days)	0	
		2x1000 mg, 14 days	2	20	
		2x1000 mg, 28 days	3	16	
	30x1000 mg	2x1000 mg, 7 days	1	16	
		2x1000 mg, 10-14 days	1 (14 days)	2	
		2x1000 mg, 14 days	1	2	
		2x1000 mg, 28 days	2	4	
Cephalexin	16x500 mg^c^	2x1000 mg, 7 days	2	4	n/a
	16x1000 mg^c^	2x1000 mg, 7 days	1	2	
Cefixime	5x400 mg^c^	1x400 mg, 10-14 days	2 (10 days)	0	n/a
		1x400 mg, 14 days	3	1	
		1x400 mg, 28 days	6	2	
	10x400 mg^c^	1x400 mg, 10-14 days	1 (10 days)	0	
		1x400 mg, 14 days	2	6	
		1x400 mg, 28 days	3	2	
Cefuroxime	10x125 mg^c^	2x500 mg, 10-14 days	8 (10 days)	0	n/a
		2x500 mg, 14 days	12	8	
		2x500 mg, 28 days	23	6	
	10x250 mg^c^	2x500 mg, 10-14 days	4 (10 days)	0	
		2x500 mg, 14 days	6	4	
		2x500 mg, 28 days	12	8	
	8x500 mg	2x500 mg, 10-14 days	3 (12 days)	0	
		2x500 mg, 14 days	4	4	
		2x500 mg, 28 days	7	0	
	10x500 mg^c^	2x500 mg, 10-14 days	2 (10 days)	0	
		2x500 mg, 14 days	3	2	
		2x500 mg, 28 days	6	4	
	12x500 mg	2x500 mg, 10-14 days	2 (12 days)	0	
		2x500 mg, 14 days	3	8	
		2x500 mg, 28 days	5	4	
	14x500 mg^c^	2x500 mg, 10-14 days	2 (14 days)	0	
		2x500 mg, 14 days	2	0	
		2x500 mg, 28 days	4	0	
	15x500 mg	2x500 mg, 10-14 days	2 (14 days)	2	
		2x500 mg, 14 days	2	2	
		2x500 mg, 28 days	4	4	
	16x500 mg^c^	2x500 mg, 10-14 days	2 (14 days)	4	
		2x500 mg, 14 days	2	4	
		2x500 mg, 28 days	4	8	
	20x500 mg	2x500 mg, 10-14 days	1 (10 days)	0	
		2x500 mg, 14 days	2	12	
		2x500 mg, 28 days	3	4	
	24x500 mg	2x500 mg, 10-14 days	1 (12 days)	0	
		2x500 mg, 14 days	2	20	
		2x500 mg, 28 days	3	16	
Norfloxacin	20x400 mg^c^	2x400 mg, 3 days	1	14	n/a

Co-amoxiclav – amoxicillin and clavulanic acid; n/a – not applicable; extra units of drugs are presented with reference to the original formulation

^a^The Intersectoral Society for Antibiotic Resistance Control (ISKRA) guidelines on antimicrobial treatment and prophylaxis of urinary tract infections

^b^International Clinical Practice Guidelines for the Treatment of Acute Uncomplicated Cystitis and Pyelonephritis in Women: A 2010 Update by the Infectious Diseases Society of America and the European Society for Microbiology and Infectious Diseases

^c^included in Croatian national reimbursement lists on 1^st^ August 2018

**Table 4:** Accordance of the package sizes of selected drugs with guidelines for urinary tract infections II

Drug	Package size	ISKRA^a^	Min No. of packages	Extra units	IDSA^b^	Min No. of packages	Extra units
Ciprofloxacin	10x250 mg^c^	2x500 mg, 7-10 days	4 (10 days)	0	2x500 mg, 7 days	3	2
		2x500 mg, 14 days	6	4			
					1000 mg, 7 days (extended release)	n/a	n/a
		2x500 mg, 28 days	12	8			
	10x500 mg^c^	2x500 mg, 7-10 days	2 (10 days)	0	2x500 mg, 7 days	2	6
		2x500 mg, 14 days	3	2			
					1000 mg, 7 days (extended release)	n/a	n/a
		2x500 mg, 28 days	6	4			
Fosfomycinum	1x2000 mg^c^	n/a			1x3000 mg	n/a	n/a
	2x2000 mg	n/a			1x3000 mg	n/a	n/a
	1x3000 mg^c^	n/a			1x3000 mg	1	0
	2x3000 mg^c^	n/a			1x3000 mg	1	1
Levofloxacin	1x500 mg	n/a			1x750 mg, 5 days	8	0.5
	5x500 mg	n/a			1x750 mg, 5 days	2	2.5
	7x500 mg	n/a			1x750 mg, 5 days	2	6.5
	10x500 mg^c^	n/a			1x750 mg, 5 days	1	2.5
	14x500 mg	n/a			1x750 mg, 5 days	1	6.5
Nitrofurantoin	30x50 mg^c^	2x100 mg, 7 days	1	2	2x100 mg, 5 days (monohydrate, macrocrystals)	1	10
Sulfametoxazole, trimethoprim	20x120 mg^c^	2x960 mg, 28 days	23	12	2x960 mg, 3 days	3	12
					2x960 mg, 14 days	12	16
	20x480 mg^c^	2x960 mg, 28 days	6	8	2x960 mg, 3 days	1	8
					2x960 mg, 14 days	3	4
	20x960 mg^c^	2x960 mg, 28 days	3	4	2x960 mg, 3 days	1	14
					2x960 mg, 14 days	2	12

n/a – not applicable; extra units of drugs are presented with reference to the original formulation

^a^The Intersectoral Society for Antibiotic Resistance Control (ISKRA) guidelines on antimicrobial treatment and prophylaxis of urinary tract infections

^b^International Clinical Practice Guidelines for the Treatment of Acute Uncomplicated Cystitis and Pyelonephritis in Women: A 2010 Update by the Infectious Diseases Society of America and the European Society for Microbiology and Infectious Diseases

^c^included in Croatian national reimbursement lists on 1^st^ of August 2018

Cefpodoxime, doxycycline, flucloxacilin, linezolid, moxifloxacin, nitroxolin, telithromycin were not identified in the included guidelines. Treatment duration for linezolid varies as 2x600 mg 10-14 days. Package sizes are 10, 20, 28 or 30 units of 600 mg tablets and only size of 10 units is under reimbursement. The largest package would result in 2 extra units. All others were in accordance with the recommendation in SmPC. Proposed treatment durations for moxifloxacin in different indications are 1x400 mg for 5-10 days, 7 days, 10 days and 14 days. We identified package sizes containing 1, 5, 7, 10, 14, 25, 28 and 30 units of 400 mg tablets of moxifloxacin. Packages of 5, 7 and 10 units of 400 mg moxifloxacin were identified in national reimbursement lists. Most packages match at least one of the recommendations. Larger packages (25, 28 and 30 units) have 11, 14 or 16 extra units. Telithromycin is approved under the centralised procedure in European Union and it used to be under national reimbursement. The SmPC of this drug recommends treatment regimens and duration of 1x800 mg for 5 days or 7-10 days depending on the indication. This study identified package sizes of 10, 14 and 20 400 mg tablets all of which were in accordance with at least one of the recommended treatment durations. Proposed treatment durations for doxycycline in different indications are 1x100-200 mg and 300 mg for 1 day, 2x100 mg for 7, 14 or 10-21 days and 1x200 mg for 7 days. We identified 100 mg doxycycline packed as 5, 6 and 25 capsules. For package sizes of 5 and 6 units there would inevitably be extra units in every case, except for treatment 2x100 mg for 10-21 days. Package containing 25 capsules of doxycycline matched with treatment for 14.5 days. SmPCs of flucloxacillin, nitroxoline and cefpodoxime did not propose treatment duration. However, SmPCs of mentioned drugs include a statement *consideration should be given to official guidance on the appropriate use of antibacterial agents and the local prevalence of resistance*. It should be noted that cefpodoxime is a third-generation cephalosporin antibiotic, therefore it may fall under scope of some of the included guidelines but recommended treatment duration was not clearly specified.

## Discussion

This study identified accordance of drug package sizes with national guidelines in 30 cases of total 104 combinations. Packages were matched in 4 out of 37 cases with IDSA guidelines and in one of total 6 cases with NICE guidelines. Although this study cross-referenced accordance of package sizes of antibiotics with national and international guidelines, accordance with local or national guidelines is of the greatest importance [20]. It is encouraging that national recommendations were met more often than NICE or IDSA recommendations. However, when referring to drug packages under reimbursement, accordance with national guidelines was present in 18 of total of 53 combinations. It is possible to provide some rationalisation for this discrepancy. Antibiotics have various indications many of which exceed the scope of this study. It is possible that drug packages under reimbursement that were not in accordance with guidelines for sore throat or urinary tract infections would match recommended treatment durations for other indications. However, a recent study in England reported that patients without comorbidity most often seek physician consultation for acute cough, sore throat and urinary tract infections [21]. Another study reported that most antibiotics in primary care setting were prescribed for respiratory and urinary tract infections, 7.7% for sore throat and 20.6% for urinary tract infections [3]. Reimbursement lists included some drugs not mentioned in the ISKRA guidelines, but mentioned in IDSA or NICE guidelines. Reimbursement lists are updated every few months and often include newly marketed drugs, while guidelines by professional societies, such as ISKRA guidelines, cannot be updated this often. It may be expected that the national reimbursement lists should be in accordance with national treatment recommendations. However, this study could not lead to this conclusion. Previous studies have found that there is low accordance of prescribed antibiotics with guidelines. In English primary care, 23% of inappropriate prescriptions were attributed to sore throat [22].

Furthermore, literature reports that less than 40% of surgeons feel very confident in planning the duration of antibiotic treatment and 31% of prescribers in acute-care hospitals lacked confidence for determining duration of therapy [23, 24]. Young physicians may rely on packages sizes, especially of those under reimbursement, to determine treatment duration. In such scenario, accordance with guidelines in some cases cannot be established. It has been previously identified that poor ability to match package size with treatment duration may be an obstacle for following guidelines in antibiotic prescribing [11]. Therefore, the relevance of this finding should be further investigated. Furthermore, sore throat has been outlined as the condition that contributed the most to inappropriate prescribing [22].

The results of this study are consistent with findings of previous studies on this topic [10, 11]. Out of 32 most common antibiotic-prescribing scenarios in Australia, there were only 4 in which the drug package size aligned with duration recommended by The Australian (electronic) Therapeutic Guidelines. McGuire et al*.* argue that since mismatch is found more commonly than match, there might be a large quantity of redundant antibiotics in the community [11]. Mukherjee et al*.* found comparable results in India and proposed revision of antibiotic package sizes by specialists, industry and health policy makers to devise steps for reducing leftover antibiotics [10].

Excess units of antibiotics may put a strain on healthcare systems in different ways. Leftover antibiotics may increase risk of self-medication with these drugs when they are not indicated and for shorter period then recommended. Unused and unnecessary drugs are a financial burden for insurance and later for pharmacies collecting pharmaceutical waste [25]. Moreover, improper disposal of excess antibiotics supports emergence of antibiotic resistance. Consumers tend to keep leftover antibiotics or dispose them in household waste and are unaware of programs for safe disposal of drugs provided free by community pharmacies [26].

Now, when there is evidence that classes of drugs other than antibiotics may promote antimicrobial resistance, the importance of rational pharmacotherapy is emphasized more than ever [27]. The results of this study contribute to the opinion that dispensing the exact number of pills may have advantage over dispensing drugs prepacked by the manufacturer when it comes to dispensing antibiotics. Previous studies have shown that dispensing the exact number of pills of antibiotics reduces number of pills to reimburse, reduces number of non-recycled pills and it has a positive impact on the environment and on adherence to therapy [28]. In the light of recent criticism of completing the course of antibiotics it should be stated that in the future shorter course treatments or even individualized antibiotic course treatments may be expected [29–31]. These may further support drug dispensing. Taking mentioned possible negative impacts that leftover antibiotics may have for healthcare system and the public, patient adherence to antibiotic therapy goes beyond individual health. Therefore, measures that promote patient adherence to therapy may become essential to programs of antibiotic resistance management. Furthermore, health-care providers should outline the importance of adherence to therapy and pharmaceutical waste disposal with patients that take antibiotics. It may be difficult to prescribe the tailor-made dosing equal to the total amount of antibiotic required for each patient with each diagnosis to be treated. This is more difficult in practices among paediatric patients where the dosage is determined by their body weight. We can estimate the total amount of antibiotic required in the course of treatment but to provide the tailor-made total amount of antibiotic, in order to limit environment contamination, is more difficult than the appropriate disposition of the leftover amount of antibiotics. Study conducted among Canadian physicians reported that only 60% of them often address topic of how to dispose of antibiotics with patients [32]. More attention should be given to the appropriate disposition of the leftover amount of antibiotics and to patient consultation on this matter.

It is ambitious to expect that package sizes of antibiotics are fully in accordance with guidelines as guidelines are often susceptible to change. It would be irrational to limit indications of antibiotics that by package size are not fully in accordance with the guidelines. However, some accordance should be mandatory, especially for drugs under reimbursement.

Great number of mismatched cases, both in general and under reimbursement, may open space for savings in healthcare systems. One of the ways this can be established is exact accordance of drug package size with the indication. However, this could backfire in terms of regulatory expenses for marketing authorisation holders. Other possible solution would be dispensing the exact number of pills but this raises the cost of pharmacy service and limits time available for patient consultation [33]. Moreover, recently the EU has increased efforts against counterfeit drugs introducing a Falsified Medicine Directive that allows verification of the authenticity of individual drug packages [34, 35]. Dispensing the drugs to final consumer would allow this verification to exist in the chain from manufacturer to the supplier, but not from pharmacy to the patient.

It should be noted that there is no room for expensive when it comes to antibiotic resistance. Every cost and contribution to restraining the emergence of antibiotic resistance is reasonable and justified as this is one battle most expensive if lost [36]. Patient outcomes should be in the focus, rather than cost containment [37].

### Limitations

In this study 100% patient adherence to the treatment was assumed but in practice patient adherence is often variable and poor [38]. Hence, the number of leftover antibiotics and possible consequences of such practice may be augmented in real-life setting. It should be stated that not all approved package sizes are available on the market. Although the study excluded drugs that are not marketed, the marketing authorisation holder may hold an approval for a number of package sizes and decide to place only one of them on the market. This information would not be visible in the drug database. This needs to be acknowledged as a limitation to the study. Due to the fact that in this study we identified drugs that were under reimbursement, drugs that are likely to be placed on the market, we believe the study has important findings. Another limitation is that although large package sizes (>30) were excluded from the study, it is possible that some package sizes intended for hospital use were included in the study if they were not specified as such in the database. Furthermore, based on the results of this study we can only assume that non-accordance of package size to the guidelines may contribute to the problem of antimicrobial resistance as further research is needed to confirm such assumptions.

## Conclusions

Leftover antibiotics may contribute to the emergence of antibiotic resistance. One of the causes of leftover antibiotics is poor accordance of antibiotic package size with treatment recommendation duration. Switch to dispensing the exact number of pills of antibiotics or regulatory incentives for mandatory accordance with guidelines would inevitably result in greater costs for healthcare system and patients. However, poor accordance of package size with guidelines should be identified as a potential target for reduction of excess antibiotics in the community. Improvements in the packaging system and health personal drug administration system are warrant. Furthermore, measures that promote patient adherence to therapy and patient education should be considered essential to manage proper handling of leftover antibiotics. Furthermore, health-care providers should discuss how to dispose of excess antibiotics with patients and the importance of not sharing leftover antibiotics.

## Abbreviations

IDSA: The Infectious Diseases Society of America
ISKRA: Intersectoral Society for Antibiotic Resistance Control
IU: International units
NICE: National Institute for Health and Care Excellence
SmPC: Summary of product characteristics
